# LINC00543 promotes colorectal cancer metastasis by driving EMT and inducing the M2 polarization of tumor associated macrophages

**DOI:** 10.1186/s12967-023-04009-6

**Published:** 2023-02-25

**Authors:** Jinsen Zheng, Rongzhang Dou, Xinyao Zhang, Bo Zhong, Chenggang Fang, Qian Xu, Ziyang Di, Sihao Huang, Zaihuan Lin, Jialin Song, Shuyi Wang, Bin Xiong

**Affiliations:** 1 Department of Gastrointestinal Surgery, Zhongnan Hospital of Wuhan University, Donghu Road 169, Wuhan, 430071 Hubei China; 2 Department of Gastric and Colorectal Surgical Oncology, Zhongnan Hospital of Wuhan University, Donghu Road 169, Wuhan, 430071 Hubei China; 3 Hubei Key Laboratory of Tumor Biological Behaviors, Donghu Road 169, Wuhan, 430071 Hubei China; 4 Hubei Cancer Clinical Study Center, Donghu Road 169, Wuhan, 430071 Hubei China; 5 Medical Research Institute of Wuhan University, Wuhan University, Wuhan, China

**Keywords:** Epithelial-mesenchymal transition, Long non-coding RNA, Metastasis, Pre-miR-506-3p transport, Tumor microenvironment (TME)

## Abstract

**Background:**

The interaction between the tumor-microenvironment (TME) and the cancer cells has emerged as a key player in colorectal cancer (CRC) metastasis. A small proportion of CRC cells which undergo epithelial-mesenchymal transition (EMT) facilitate the reshaping of the TME by regulating various cellular ingredients.

**Methods:**

Immunohistochemical analysis, RNA immunoprecipitation (RIP), RNA Antisense Purification (RAP), dual luciferase assays were conducted to investigate the biological function and regulation of LINC00543 in CRC. A series in vitro and in vivo experiments were used to clarify the role of LINC00543 in CRC metastasis.

**Results:**

Here we found that the long non-coding RNA LINC00543, was overexpressed in colorectal cancer tissues, which correlated with advanced TNM stage and poorer prognosis of CRC patients. The overexpression of LINC00543 promoted tumorigenesis and metastasis of CRC cells by enhancing EMT and remodeling the TME. Mechanistically, LINC00543 blocked the transport of pre-miR-506-3p across the nuclear-cytoplasmic transporter XPO5, thereby reducing the production of mature miR-506-3p, resulting in the increase in the expression of FOXQ1 and induction of EMT. In addition, upregulation of FOXQ1 induced the expression of CCL2 that accelerated the recruitment of macrophages and their M2 polarization.

**Conclusions:**

Our study showed that LINC00543 enhanced EMT of CRC cells through the pre-miR-506-3p/FOXQ1 axis. This resulted in the upregulation of CCL2, leading to macrophages recruitment and M2 polarization, and ultimately stimulating the progression of CRC.

**Supplementary Information:**

The online version contains supplementary material available at 10.1186/s12967-023-04009-6.

## Introduction

According to the global cancer statistics from 2020, colorectal cancer (CRC) ranked third in terms of cancer incidence and second in terms of cancer mortality [1]. Metastasis remains the major cause of death in CRC patients [2]. The tumor microenvironment (TME) is not a silent spectator, but rather an active participant that promotes tumor metastasis [3]. Tumor-associated macrophages (TAMs), one of the main cell types in the TME, are commonly activated and polarized to M1/M2 state [4]. The M2 macrophages are known to play an anti-inflammatory and cancer-promoting role during tumor progression [5, 6]. Cancer cells promote the recruitment of macrophages and their M2 polarization to build an immunosuppressive TME, which in turn facilitate cancer progression [7, 8]. Since the cross-talk between the TAMs and tumors has been largely reported, many of the recent studies have considered TAMs as promising therapeutic targets for cancer [9]. However, the underlying molecular mechanism regulating TAMs recruitment and polarization in CRC is still unclear.

Epithelial mesenchymal transition (EMT) is an important pathophysiological process in tumor progression [10] during which the cancerous cells gain the capacities of migration and invasion by losing their polarity and inter-cellular junctions [11–14]. In addition, many previous studies have uncovered that cancer cells undergoing EMT secrete various cytokines to reshape the tumor immune microenvironment [15]. In melanoma, cancer cells undergoing EMT have been reported to produce TGF-β and TSP-1 which repressed the anti-cancer immune response that accelerated cancer metastasis [16]. Hepatocellular carcinoma cells that underwent EMT secreted CCL2 to recruit TAMs that facilitated tumor metastasis [17]. Therefore, exploring how tumor cells that underwent EMT could regulate the immune microenvironment in CRC is important to identify novel therapeutic targets for treating CRC metastasis.

Long non-coding RNAs (lncRNAs) are non-protein coding RNA transcripts that play vital roles in tumor progression, and metastasis [18, 19]. With regards to CRC, lncRNAs have been recognized as important participants in the regulation of EMT and the TME. For instance, Yue, Ben et al. revealed that a positive feedback loop between the lncRNA CYTOR and the Wnt/β-Catenin axis promoted EMT and metastasis of colorectal cancer cells [20]. Wu Nan and colleagues reported that *LINC00941* was directly bound to SMAD4 protein, which induced EMT and enhanced colorectal cancer metastasis [21]. Reciprocally, Liang, Zhen-Xing, and colleagues elucidated the mechanism of lncRNA RPPH1, which promoted the M2 polarization of TAMs and accelerated colorectal cancer metastasis [22]. The aberrant expression of *LINC00543* was reported to be correlated with immunosuppression and poorer prognosis of CRC patients [23]. However, the exact mechanism underlying the regulation of EMT and the TME by *LINC00543* in CRC is still unknown.

In this study, we found that *LINC00543* was significantly elevated and markedly correlated with advanced TNM stage and poorer overall survival (OS) in colorectal cancer patients. In vitro experimental data showed that high *LINC00543* expression activated EMT and boosted TAMs recruitment and M2 polarization, thereby accelerating the progression and metastasis of CRC. The above results indicated the oncogenic role of *LINC00543*. Further mechanistic exploration revealed that *LINC00543* inhibited the transport of pre-miR-506-3p by the nuclear-cytoplasmic transporter XPO5 and downregulated the expression of miR-506-3p, thereby upregulating FOXQ1 expression and inducing EMT. In addition, upregulated FOXQ1 in tumor cells promoted the secretion of CCL2, which recruited TAMs and promoted their M2 polarization. And we verified the function of *LINC00543* in regulating EMT and macrophage polarization to promote the metastasis of colorectal cancer in nude mice. The above findings revealed a novel mechanism of *LINC00543* regulating the miRNA maturation process and metastasis of CRC.

## Materials and methods

### Patients and tissue samples

We collected primary tumor tissues and paired normal colorectal tissues (> 5 cm distance from the cancer tissue margin) from 40 CRC patients from the Zhongnan Hospital of Wuhan University (Wuhan, China). All enrolled patients were definitively diagnosed with colorectal adenocarcinoma by histopathology, and none had received neoadjuvant radiotherapy or/and chemotherapy before undergoing resection. In addition, all the enrolled patients in the group had corresponding available survival data. This study was endorsed by the Ethics Committee of Zhongnan Hospital of Wuhan University in compliance with the Declaration of Helsinki. Informed consent was obtained from all the patients before sample collection.

### RNA antisense purification (RAP)

RAP analysis was performed in accordance with the manufacturer’s instructions for the RNA Antisense Purification (RAP) kit (BersinBion, Bes5103-1, China). Briefly, 4 × 10^7^ cells per group were collected and added to the lysis solution for cell lysis. After DNA removal, the LINC00543 biotin probe, the LINC00543 segmental biotin probe, and the LINC00543 segmental antisense nucleotide fragment biotin probes were co-incubated at 37 °C for 180 min. Next, the mixture of probes and samples were added to the streptavidin magnetic beads and incubated on a vertical mixer at room temperature for about 30 min. After removing the proteins by proteinase K, the RNA was distributed for RT-PCR and agarose gel electrophoresis analysis. The sequences of the probes used for RAP are shown in the Supplementary Table S4.

### RNA binding protein immunoprecipitation (RIP)

RIP analysis was performed using the Magna RIP RNA-Binding Protein Immunoprecipitation Kit (Millipore, MA, USA). We first collected 2 × 10^7^ cells that had been washed with ice-cold PBS. The configured RIP lysis solution was mixed and lysed at 0 °C for 5 min. DNA was then removed, and the nucleotide fragments of LINC00543 segments or antisense nucleotide fragments were added to hybridize at 37 °C for 3 h. The samples were then mixed with anti-XPO5 antibody and protein A/G magnetic beads and incubated on a vertical mixer at 4 °C for 12 h. The proteins were then digested after washing away the unbound material with RIP wash buffer. Finally, the RNA was extracted for subsequent experiments. The sequences of the probes used for RIP are shown in the Additional file 1: Table S4.

### Flow cytometry

Macrophages and cells extracted from the tumors of nude mice were made into single-cell suspensions. The suspensions were mixed with antigen specific fluorescent antibodies and incubated on ice for 2 h (PE Mouse anti-Human CD163, APC Mouse anti-Human CD86, FITC Mouse anti-Human CD206, PerCPMouse anti-Human CD80, all from Biolegend, USA). Cells were washed twice by adding 3 ml of the flow buffer and were then resuspended in 600 μl of the flow buffer. Flow cytometry was performed using the FACSCalibur flow cytometer (BD Biosciences, USA). Flow cytometry results were analyzed using the FlowJo software (FlowJo, USA).

### Statistics

The data were expressed as mean ± standard error (m ± SE) and were analyzed by two-tailed Student’s t-test. All experiments were performed in triplicate and statistical significance was defined as p < 0.05 using the GraphPad Prism software (version 6.0, GraphPad Software, USA) for Windows.

## Results

### *LINC00543* is highly expressed in CRC tissues and is associated with poor prognosis of CRC patients

**Fig. 1:**
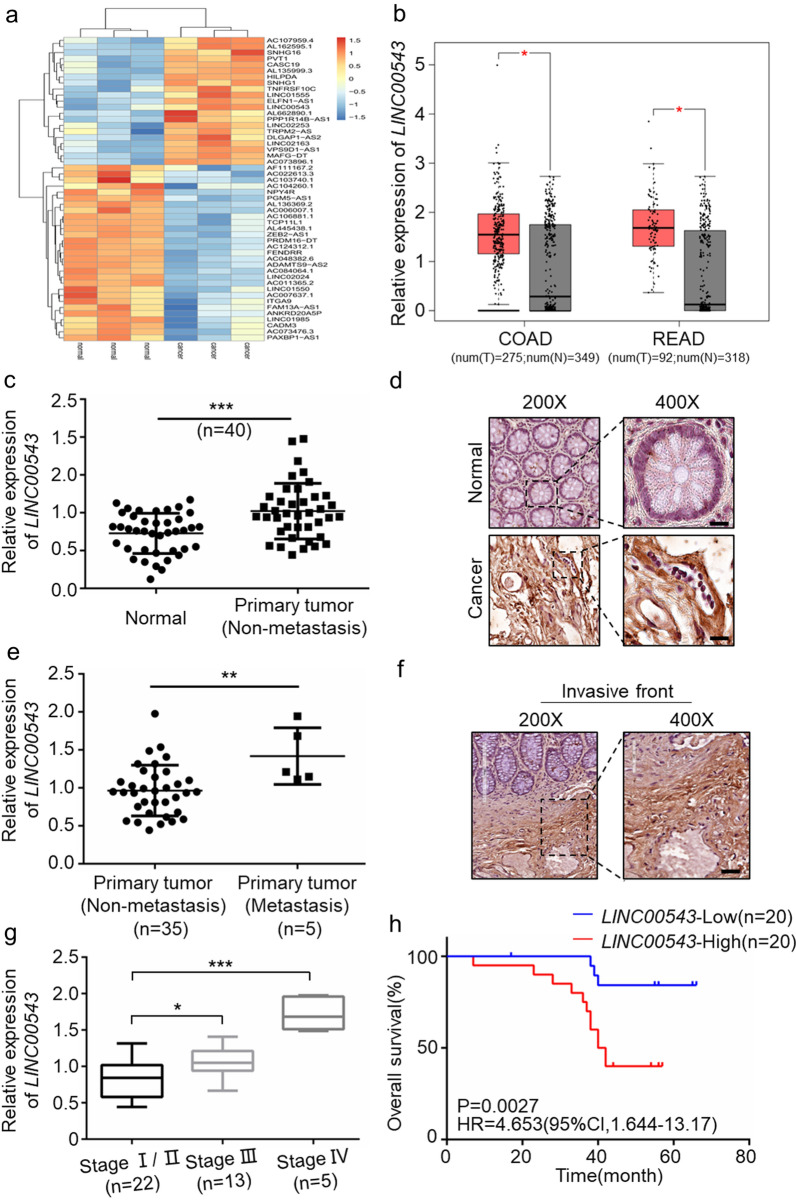
*LINC00543* is an oncogene in CRC. **a** The expression of *LINC00543* in CRC Gene Chips (GES110715). **b** The expression of *LINC00543* through online analysis website GEPIA2. **c** Relative expression of *LINC00543* detected by qRT-PCR in 40 CRC primary tumor tissues and their paired normal tissues normalized to 18S. **d** In situ hybridization (ISH) analysis of *LINC00543* expression in colorectal cancer tissues and their matched normal tissues (scale bar = 100 μm). **e** Comparison of *LINC00543* expression in samples from CRC patients with metastatic colorectal cancer and patients without metastasis. **f** The expression of *LINC00543* in the invasive fronts (scale bar = 100 μm). **g** The relationship between the expression levels of *LINC00543* and TNM stages in 40 CRC patients. **h** The database from Kaplan–Meier Plotter showed the relationship between the expression of *LINC00543* and the overall survival rate. P < 0.001

Based on the analysis of the datasets from the GEO database (GES110715) and the online analysis website GEPIA2 (http://gepia2.cancer-pku.cn/#index), we found that the expression of *LINC00543* was higher in the CRC tissues as compared with the normal colorectal tissues (Fig. 1a and b). Then, we detected the expression of *LINC00543* in 40 CRC tissues and their matched normal tissues, and the results were consistent with the results from the bioinformatics analysis. Besides, we found that the expression of *LINC00543* was higher in the primary tumor tissues of CRC patients with distant metastasis than those without distant metastasis. Notably, the expression of *LINC00543* in the invasive fronts was higher than in other regions of the primary tumor (Fig. 1c–f, Additional file 1: Fig. S1a). Moreover, we investigated the clinical data from 40 patients and found that the elevated expression of *LINC00543* was correlated with advanced TNM stage and poor prognosis (Fig. 1g and h, Additional file 1: Table S1). Also, the encoding potential and location of *LINC00543* were also analyzed, and the results showed that the *LINC00543* gene on chromosome 13 lacked the ability to encode proteins (Additional file 1: Fig. S1b–d).

The above data suggested that high *LINC00543* expression was closely related to the advanced TNM stage and poor prognosis of CRC patients.

### *LINC00543* promotes the invasion and metastasis of CRC cells

**Fig. 2:**
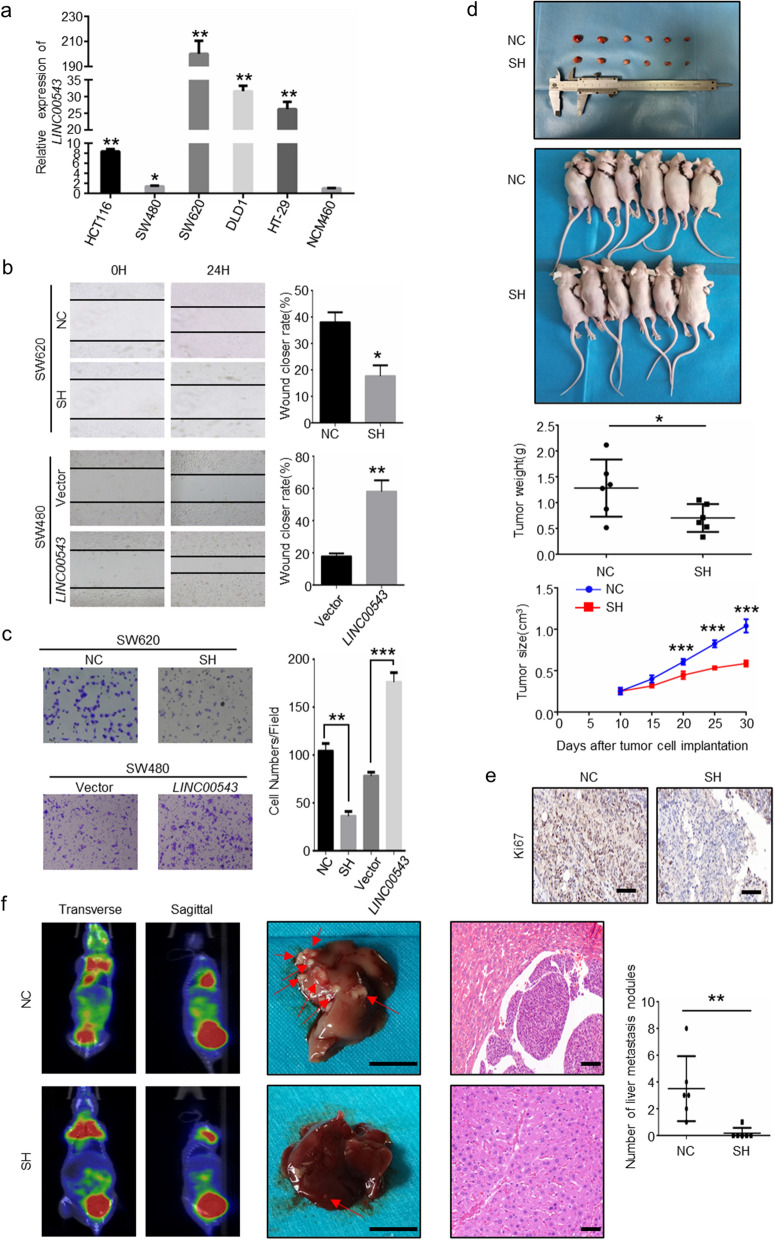
*LINC00543* promotes the invasion and metastasis of CRC cells in vitro and in vivo. **a** The relative expression of *LINC00543* in non-cancer cells NCM460 and CRC cells (HCT116, SW480, SW620, DLD1, HT-29). **b** Wound-healing assays and **c** transwell assays in SH cells and *LINC00543* cells (magnification, × 200). **d** The tumor volumes in nude mice from the NC and SH groups. **e** The immunohistochemistry analysis of tumor from the NC and the SH groups (scale bar = 100 μm). **f** PET-CT images, liver anatomy (scale, 1 cm), H&E staining of the liver, and the number of liver surface metastases in spleen-injected liver metastasis models in nude mice, in the NC and SH groups (scale bar = 100 μm)

To further explore the function of *LINC00543* in CRC, we evaluated the expression level of *LINC00543* in a normal colorectal cell line (NCM460) and five CRC cell lines (HCT116, SW480, SW620, DLD1, HT-29). The expression of *LINC00543* was significantly elevated in the CRC cell lines. Interestingly, *LINC00543* expression in metastatic CRC cell line SW620, was considerably higher than that in primary CRC cell line SW480, further indicating that *LINC00543* expression was associated with distant metastasis (Fig. 2a). We selected SW620 and SW480 to construct *LINC00543* knockdown (SH) and overexpression (*LINC00543*) cell lines separately by using lentivirus and the transfection efficiency was evaluated by qRT-PCR (Additional file 1: Fig. S2a–c). The knockdown of *LINC00543* suppressed the invasion and migration of CRC cells, while the overexpression of *LINC00543* promoted the invasion and migration of CRC cells (Fig. 2b and c). Furthermore, the tumorigenic effect of *LINC00543* was evaluated in vivo. Tumor formation and growth were inhibited upon the implantation of cell with the knockdown of *LINC00543* (Fig. 2d and e, Additional file 1: Fig. S2d). Next, we evaluated the in vivo role of *LINC00543* in metastasis by injecting CRC cells in the spleen to establish a liver metastatic mouse model. Compared with the NC group, mice in the SH group showed a lower rate of liver metastasis (Fig. 2f).

### LINC00543 regulates EMT in CRC cells

**Fig. 3:**
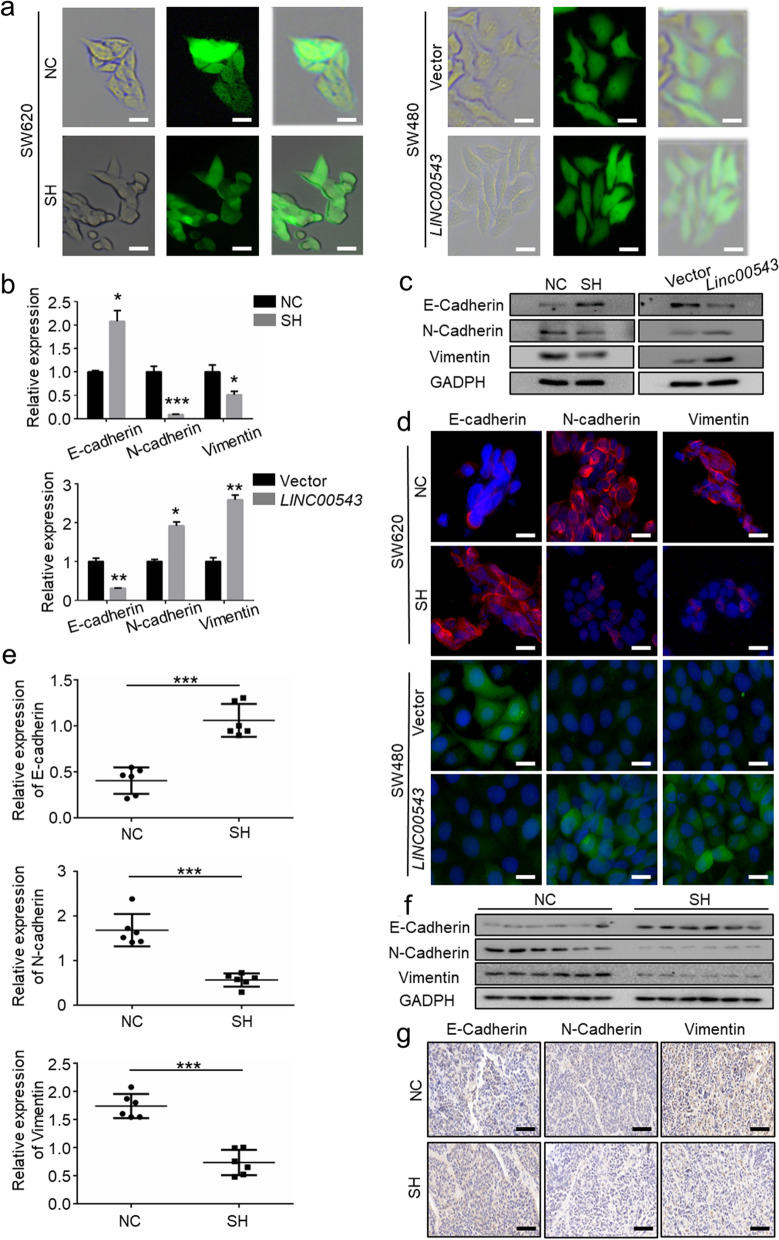
*LINC00543* promotes EMT in CRC cells. **a** The morphologies of CRC cells in response to *LINC00543* manipulation (scale bar = 25 μm) **b** qRT-PCR and **c** western blot analysis of E-cadherin and Vimentin in the SH and *LINC00543* cells. **d** E-cadherin, N-cadherin, and Vimentin expression in the SH and *LINC00543* cells were determined by immunofluorescence staining (scale bar = 25 μm). **e** and **f** The expression of EMT-related markers in the NC and SH group mice. **g** The expression of EMT-related markers in the NC and SH group mice was determined by immunohistochemistry (IHC) (scale bar = 100 μm)

Considering that EMT is a crucial process in tumor metastasis [15], we further interrogated whether *LINC00543* promoted the EMT process. We observed that in comparison with the control group, cells with the knockdown of *LINC00543* lost their typical spindle-shaped morphology, while after the overexpression of the *LINC00543*, the cells regained their spindle shape morphology (Fig. 3a). Consistently, the expression of the epithelial cell marker E-cadherin decreased in the CRC cells overexpressing *LINC00543*, whereas the expression of mesenchymal cell markers N-cadherin and Vimentin were elevated. In addition, contrary results were observed in cells overexpressing the *LINC00543* (Fig. 3b–d). In vivo, the knockdown of *LINC00543* downregulated the expression of E-cadherin and elevated the expression of N-cadherin and Vimentin (Fig. 3e and f). These observations were consistent with the immunohistochemistry (IHC) results (Fig. 3g, Additional file 1: Fig. S3).

### *LINC00543* regulates EMT by upregulating FOXQ1 in CRC cells

**Fig. 4:**
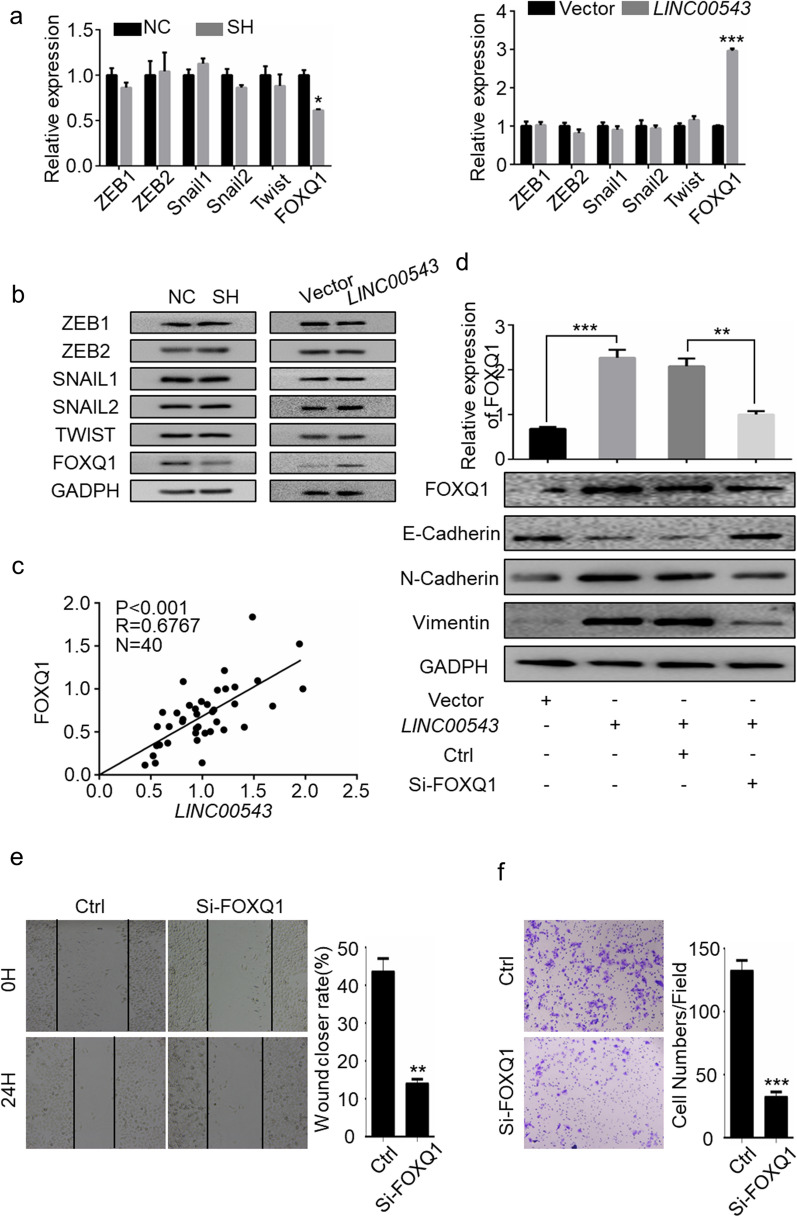
FOXQ1 is essential in the process of *LINC00543* mediated regulation of EMT in CRC cells. **a** qRT-PCR and **b** western blot analysis of EMT-related transcription factors in the SH and *LINC00543* cells. **c** Correlation between the expression of FOXQ1 and *LINC00543* in 40 patients with colorectal cancer. **d** The expression of FOXQ1 and EMT markers were detected in the *LINC00543* cells with Si-FOXQ1 transfected. **e** Wound-healing assays and **f** Transwell assays in *LINC00543* cells transfected with Si-FOXQ1 and control; magnification, × 200

In order to further uncover the molecular mechanism of *LINC00543* in EMT, we analyzed the expression of EMT-related transcription factors [24]. The results showed that the expression of FOXQ1 was significantly downregulated in cells with *LINC00543* knockdown, while it was elevated in the cells overexpressing *LINC00543*. There were no significant changes in the expression levels of other EMT-related transcription factors (ZEB1, ZEB2, Snail1, Snail2, and Twist) (Fig. 4a and b, Additional file 1: Fig. S4). To extend the correlation of our findings, we analyzed the expression of *LINC00543* and FOXQ1 in tissue specimens from 40 CRC patients and found a positive correlation between them (Fig. 4c). Moreover, the knockdown of FOXQ1 significantly attenuated the induction of EMT, invasion, and migration in CRC cells overexpressing the *LINC00543* (Fig. 4d–f).

Collectively, FOXQ1 was found to be essential in mediating the effects of *LINC00543* on EMT in CRC cells.

### *LINC00543* indirectly regulates FOXQ1 by downregulating miR-506-3p

**Fig. 5:**
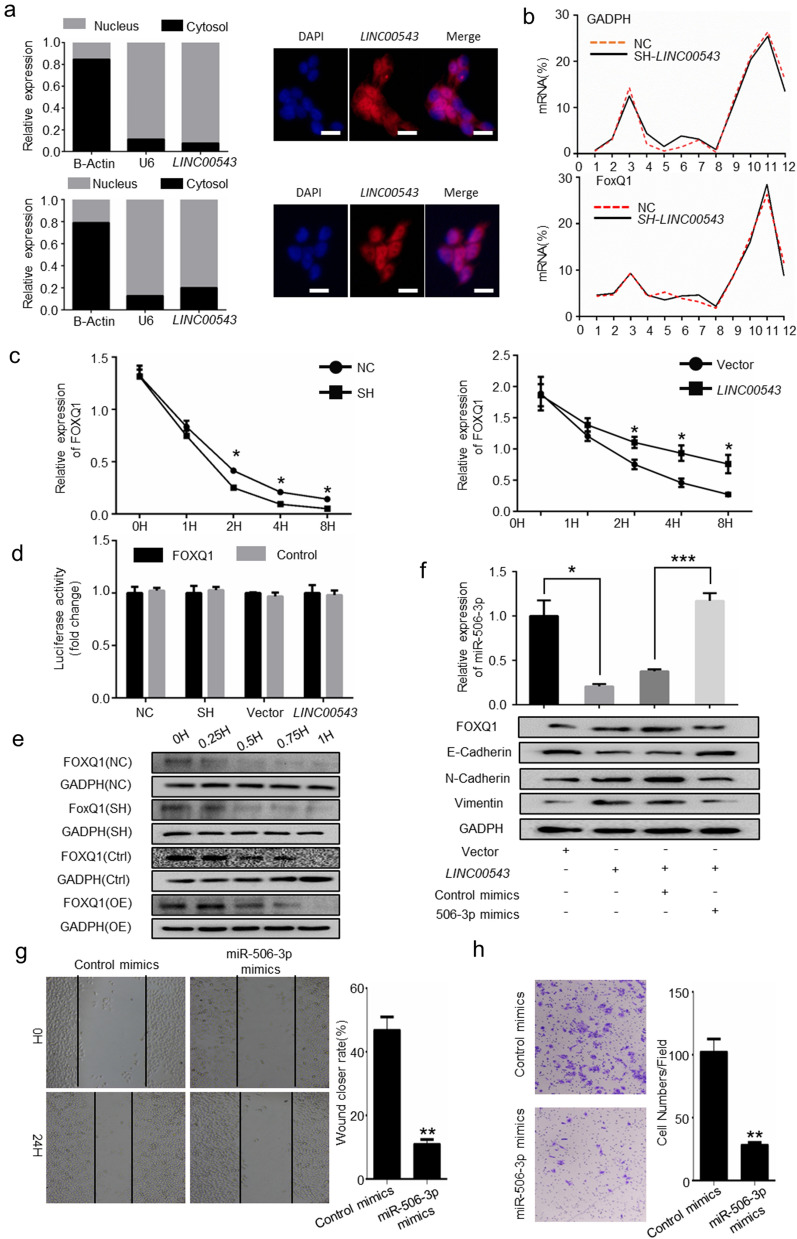
*LINC00543*/miR-506-3p/FOXQ1 regulates EMT of CRC cells. **a** Intracellular localization of *LINC00543* was identified by nuclear/cytoplasmic fractionation experiments and FISH analysis (scale bar = 25 μm). **b** Polysome Fractionation analysis evaluating the effect of *LINC00543* on FOXQ1 translation. **c** Actinomycin D analysis of the effect of *LINC00543* on FOXQ1 mRNA degradation. **d** Dual luciferase analysis of the effect of *LINC00543* on FOXQ1 DNA transcription. **e** Cycloheximide-chase analysis of the effect of *LINC00543* on FOXQ1 protein degradation. **f** The expression of FOXQ1 and EMT markers were detected in *LINC00543* cells upon treatment with miR-506-3p mimics. **g** Wound-healing assays and **h** transwell assays in *LINC00543* cells transfected with miR-506-3p mimics and control mimics; magnification, × 200

To further study the molecular mechanism of *LINC00543* regulating FOXQ1, firstly, we identified the localization of *LINC00543* in cells by fluorescence in situ hybridization and subcellular fraction analyses. The results showed that *LINC00543* was mainly localized in the cell’s nucleus (Fig. 5a). We reasoned that *LINC00543* might regulate the transcription of FOXQ1, and thus constructed a dual-luciferase reporter plasmid containing the promoter region of FOXQ1. Surprisingly, overexpression/knockdown of the *LINC00543* had no effect on the transcription of FOXQ1. Then, actinomycin D assays were conducted, and the results indicated that *LINC00543* suppressed the degradation of FOXQ1 mRNA. Additionally, based on the results from the polysome fractionation assay and cycloheximide-chase assay, we determined that *LINC00543* regulated FOXQ1 by suppressing the degradation of FOXQ1 mRNA (Fig. 5b–e, Additional file 1: Fig. S5). Our previous studies showed that miR-506-3p regulated FOXQ1 by enhancing the degradation of FOXQ1 mRNA [25], accordingly, qRT-PCR analysis was conducted to examine the expression of miR-506-3p in CRC cells overexpressing *the LINC00543*. The results indicated that miR-506-3p expression was significantly downregulated in CRC cells overexpressing the *LINC00543* (Fig. 5f). After transfecting miR-506-3p mimics into cells overexpressing *LINC00543*, we observed the positive effects of *LINC00543* overexpression on EMT, invasion and migration in CRC cells were restored (Fig. 5f, g).

These results showed that *LINC00543* indirectly regulated FOXQ1 by downregulating miR-506-3p, thereby affecting EMT, invasion and migration of colorectal cancer cells.

### *LINC00543* inhibits the transportation of pre-miR-506-3p

**Fig. 6:**
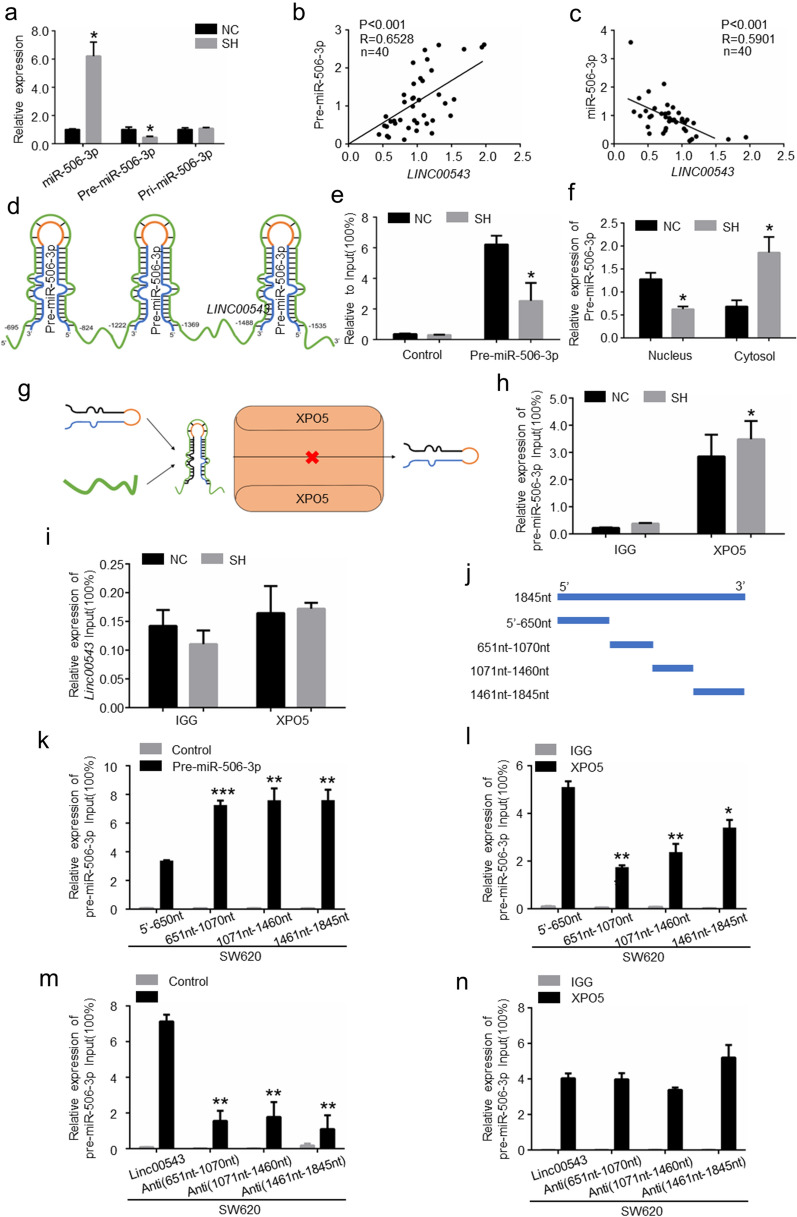
*LINC00543* blocks the maturation of miR-506-3p. **a** The expression of miR-506-3p, pre-miR-506-3p, and ppi-miR-506-3p in SH cells. **b** The relationship between the expression of *LINC00543* and Pre-miR-506-3p. **c** The relationship between the expression of *LINC00543* and the expression of miR-506-3p. **d** The binding regions of *LINC00543* to pre-miR-506-3p. **e** RAP analysis of the expression of pre-miR-506-3p in SH cells. **f** The expression of pre-miR-506-3p in the nucleus and the cytoplasm in SH cells. **g** Illustrative model showing that *LINC00543* blocked the transportation of pre-miR-506-3p. **h** RIP analysis of the interaction between XPO5 and pre-miR-506-3p in SH cells. **i** RIP analysis of the interaction between XPO5 and *LINC00543* in SH cells. **j** Schematic diagram of biotin segment probes. **k** RAP analysis of the interaction between four segments of *LINC00543* and pre-miR-506-3p. **l** RIP analysis of the interaction between XPO5 and pre-miR-506-3p in cells incubated with antisense nucleotide biotin probes of the three binding regions. **m** and **n** The interaction between *LINC00543*, XPO5, and pre-miR-506-3p were analyzed in cells incubated with the antisense nucleotide biotin probes of the three binding regions

Many studies have shown that lncRNAs regulated gene expression by competing with endogenous miRNAs [26–29]. However, the online prediction website showed that there was no direct conjunction between *LINC00543* and miR-506-3p (Additional file 1: Fig. S6a, b). Previous studies have reported that lncRNAs regulates the processing of pre-miRNA and pri-miRNA [30, 31]. Moreover, the expression of pre-miR-506-3p was significantly reduced in *LINC00543* knockdown cells, while the opposite was applicable in *LINC00543* overexpressing cells. However, the expression of pri-miR-506-3p was not significantly altered. In *LINC00543* overexpressing cells, the opposite results were obtained (Fig. 6a, Additional file 1: Fig. S7a). We also confirmed that *LINC00543* expression was positively correlated with pre-miR-506-3p expression and negatively correlated with miR-506-3p expression in 40 CRC patient tissues (Fig. 6b and c). We reasoned that *LINC00543* blocked the processing of pre-miR-506-3p to miR-506-3p.

To further investigate the mechanism of *LINC00543* acting on pre-miR-506-3p, we predicted the interaction between *LINC00543* and pre-miR-506-3p through the bioinformatics program miRanda. The results showed that three regions of *LINC00543* were directly bound to pre-miR-506-3p (Fig. 6d, Additional file 1: Fig. S6c). Next, we confirmed such prediction through RNA Antisense Purification (RAP) (Fig. 6e, Additional file 1: Fig. S7b). We know that lncRNAs can regulate the expression of miRNAs by blocking the transportation of pre-miRNA [32]. After the knockdown of *LINC00543*, the expression of pre-miR-506-3p was reduced in the nucleus but elevated in the cytoplasm (Fig. 6f), while *LINC00543* overexpression increased the expression of pre-miR-506-3p in the nucleus but downregulated its expression in the cytoplasm (Additional file 1: Fig. S7c). Therefore, we speculated that the *LINC00543* blocked the nuclear shuttle of Pre-miR-506–30 to the cytoplasm. Okada, Chimari et al**.** showed that XPO5 was a transporter of pre-miRNA from the nucleus to the cytoplasm [33] (Fig. 6g). RNA Binding Protein Immunoprecipitation showed that knocking down the *LINC00543* enhanced the transport efficiency of XPO5 for Pre-miR-506-3p (Fig. 6h), and the overexpression of *LINC00543* had the opposite effects (Additional file 1: Fig. S7d). However, XPO5 did not bind directly to the *LINC00543* and was not regulated by it. (Fig. 6i, Additional file 1: Fig. S7e and f). The analysis of agarose electrophoresis assay showed similar results (Additional file 1: Fig. S7g). These data suggested that *LINC00543* inhibited the transportation of pere-miR-506-3p by XPO5.

In order to further unveil the specific region where *LINC00543* interacted with the pre-miR-506-3p, we constructed four biotin-labelled *LINC00543* nucleotide fragment probes (Fig. 6j). RAP results showed that three regions (651nt-1070nt, 1071nt-1460nt, 1461nt-1845nt) of the *LINC00543* directly bound to the pre-miR-506-3p, and all three regions blocked the transport of pre-miR-506-3p (Fig. 6k–n, Additional file 1: Fig. S7h-k). In conclusion, *LINC00543* directly bound to pre-miR-506-3p and inhibited the transportation of pre-miR-506-3p by XPO5.

### *LINC00543* promotes macrophage recruitment and M2 polarization

**Fig. 7:**
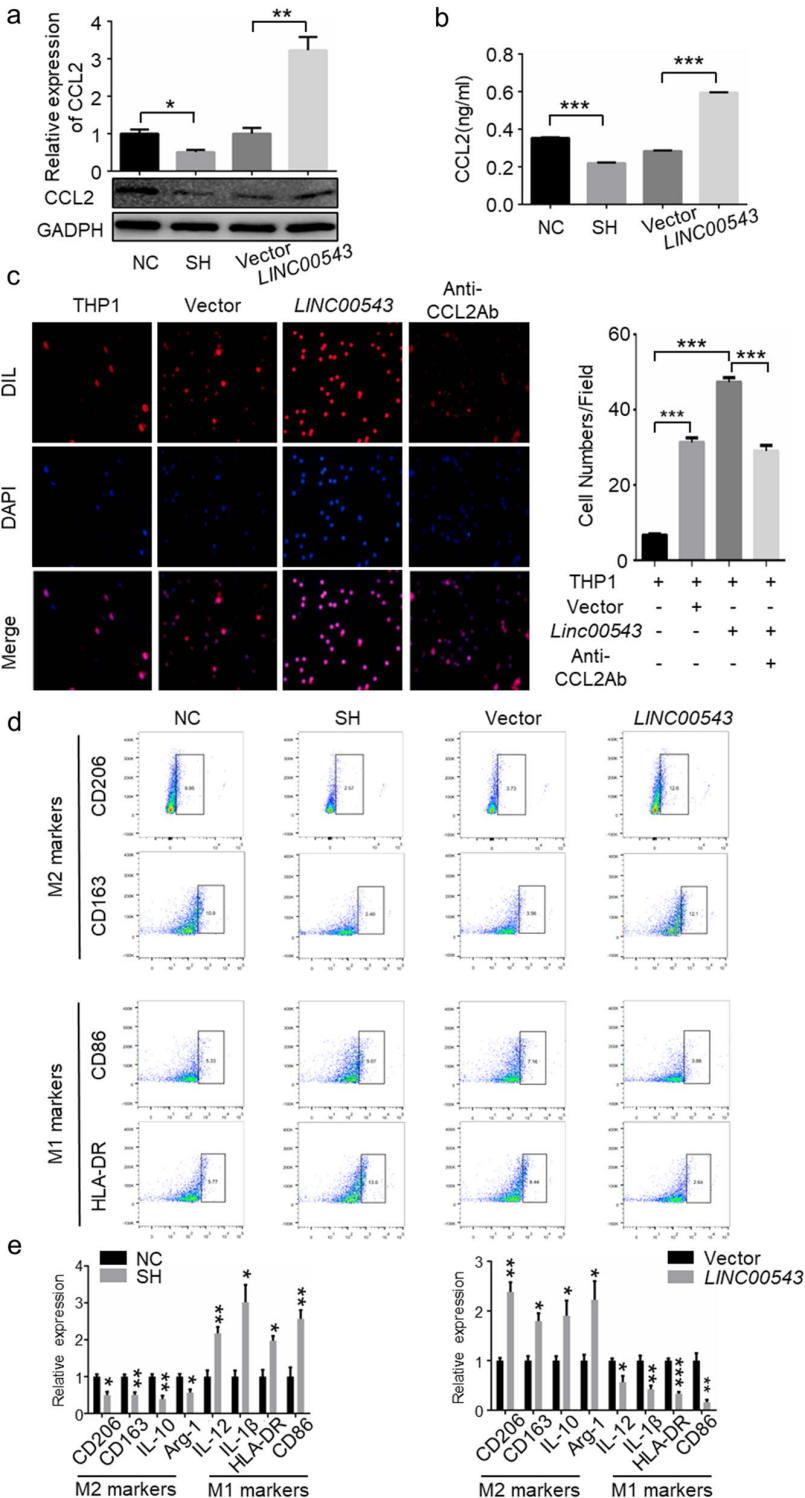
*LINC00543* promotes macrophage recruitment and M2 polarization by regulating CCL2 expression. **a** The expression of *LINC00543* in SH and *LINC00543* cells. **b** The expression of CCL2 in the supernatant of SH and *LINC00543* cells. **c** Transwell assays analyzing the effect of *LINC00543* on CCL2 mediated macrophage recruitment; magnification, × 200. **d** Flow cytometry analysis of the effect of *LINC00543* in macrophage polarization. **e** qRT-PCR analysis of M1 and M2 macrophage markers in the SH and *LINC00543* cells

It is well known that CCL2 is a chemokine which is positively regulated by FOXQ1 to promote macrophage recruitment and M2 polarization [17, 25, 34]. We found that the knockdown of *LINC00543* inhibited CCL2 expression in CRC cells and its overexpression promoted CCL2 expression (Fig. 7a). The results from ELLSA assays with the supernatant from CRC cell lines were consistent with the above findings (Fig. 7b). We co-cultured four particular cell lines (SW620-NC, SW620-SH, SW480-Vector, and SW480-*LINC00543*) with PMA-treated THP1 cells, respectively. It was found that the knockdown of *LINC00543* inhibited the recruitment and M2 polarization of macrophages. However, the opposite results were obtained with *LINC00543* overexpressing cells (Fig. 7c–e, Additional file 1: Fig. S8). Overall, *LINC00543* promoted macrophage recruitment and M2 polarization by upregulating CCL2 expression.

### LINC00543 promotes tumorigenesis in vivo

**Fig. 8:**
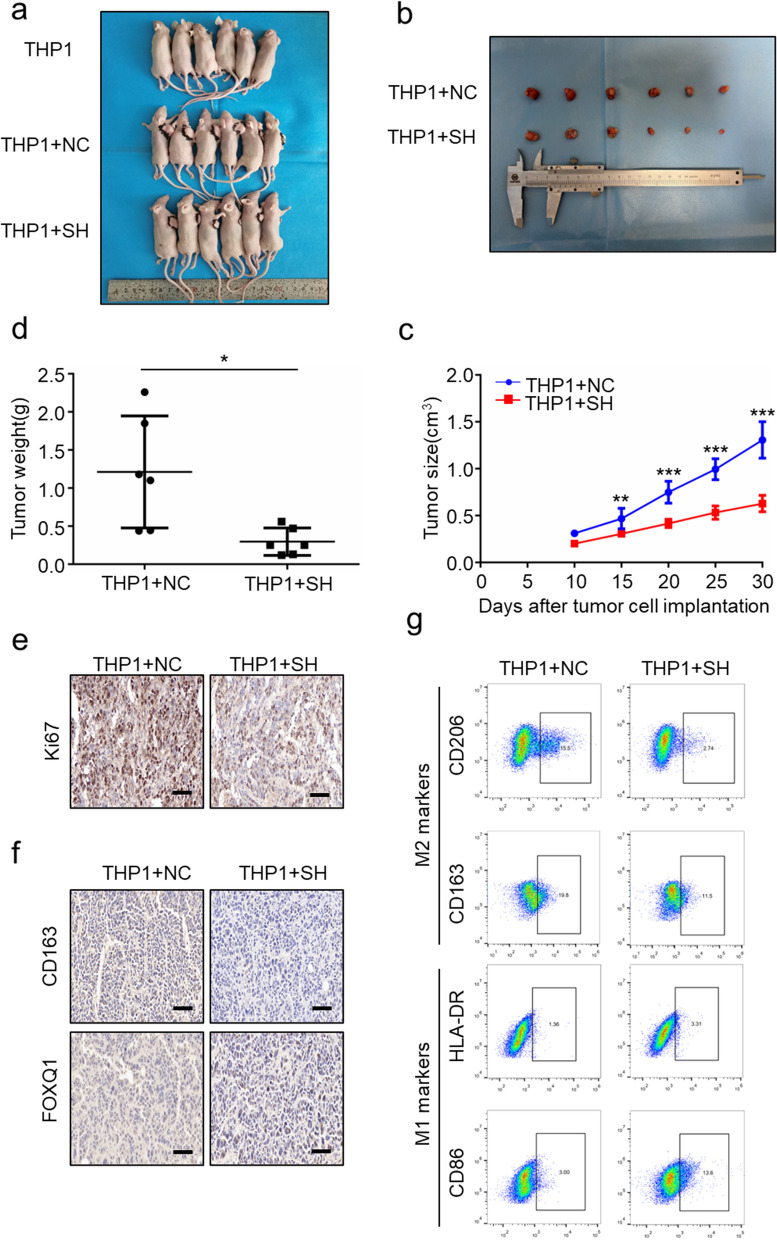
*LINC00543* promotes the CRC tumorigenesis and the M2 polarization of macrophages in vivo. **a**–**d** The tumor volumes and weights in the THP1 group, THP1 + NC group, and THP1 + SH group. **e** and **f** The immunohistochemistry analysis results of tumors from the THP1 + N and THP1 + SH groups (scale bar = 100 μm). **g** The proportion of M1 and M2 macrophages in the THP1 + NC and THP1 + SH groups

We mixed the NC group and the SH group cells with PMA-treated THP1 cells, respectively and subcutaneously injected the mixed cells into nude mice. The results showed that the knockdown of *LINC00543* inhibited CRC tumorigenesis in vivo (Fig. 8a–e, Additional file 1: Fig. S9a). The IHC results indicated that the expression levels of CD163 and FOXQ1 in xenografts from the SH group mice were downregulated compared with NC group (Fig. 8f, Additional file 1: Fig. S9b). Flow cytometry analysis of the xenografts showed that the knockdown of *LINC00543* suppressed the M2-like TAMs proportion (Fig. 8g). The results showed that *LINC00543* regulated M2 polarization of the TAMs and promoted CRC tumorigenesis in vivo.

## Discussion

**Fig. 9:**
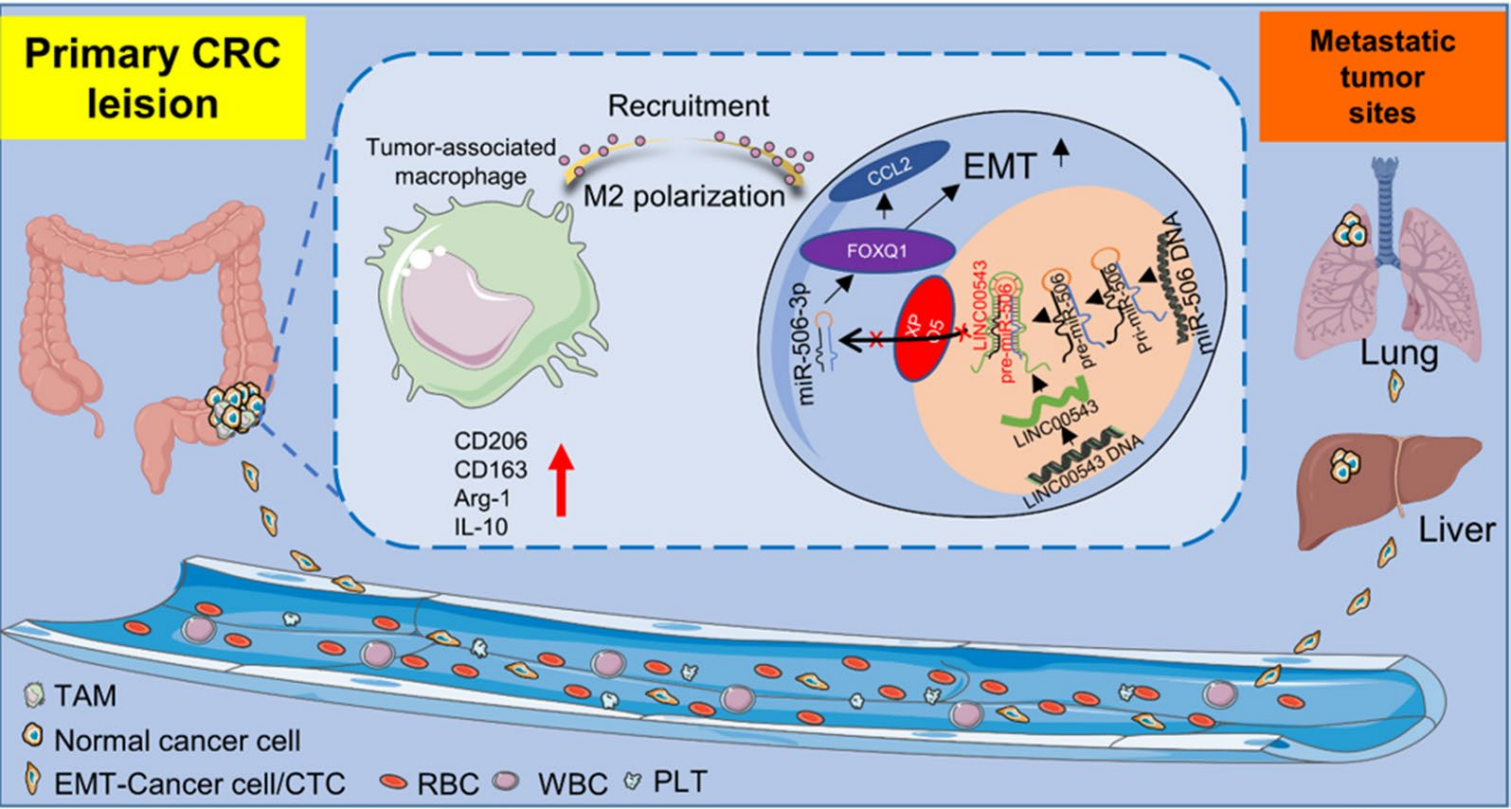
*LINC00543* promotes colorectal cancer metastasis by driving epithelial-mesenchymal transition and remodeling the tumor microenvironment

CRC is the most common gastrointestinal malignancy, and once distant metastasis occurs, the 5-year survival rate of patients decreases dramatically [35]. Therefore, it is essential to explore the metastasis associated mechanisms of CRC in greater depth and identify novel and effective targets to develop new treatment options. Increasing number of studies have shown that lncRNAs were differentially expressed in various cancers and played essential roles in cancer metastasis [36–39]. In this study, we found that *LINC00543* was highly expressed in CRC tissues and was strongly associated with metastasis and poor prognosis in CRC patients. The mechanism of lncRNAs largely depends on their subcellular localization. We found *LINC00543* was mainly localized in the nucleus and bound to the pre-miR-506-3p, preventing XPO5 from transporting pre-miR-506-3p from the nucleus to the cytoplasm, thereby inhibiting miR-506-3p expression and elevating FOXQ1 expression. Next, we showed that FOXQ1 promoted EMT and the secretion of CCL2 by CRC cells. Moreover, the upregulation of CCL2 secretion recruited TAMs and induced their polarization to the M2 phenotype, which ultimately promoted CRC progression (Fig. 9).

EMT is a complex biological program that is extensively involved in cancer development and metastasis [10]. Many of the current studies have reported that EMT activated the stemness of gastric cancer cells and mediated chemoresistance [40, 41]. In addition, increasing number of interesting studies have reported that tumor cells undergoing EMT promoted cancer metastasis by remodeling the TME. These studies mainly revolved around the modulation of the immune cells in the TME. Among them, TAMs attracted much attention because of their indispensable role in cancer progression. The miR-195-5p/NOTCH2 axis has been reported to induce EMT in CRC cells, which promoted IL-4 secretion to enhance TAMs polarization, thereby promoting tumor progression [42]. High ZEB1 expression accelerated cervical cancer progression through CCL8-induced TAMs recruitment [43]. Here, we observed that the *LINC00543*/pre-miR-506-3p/FOXQ1 axis induced EMT in CRC cells which enhanced the CCL2-mediated macrophage recruitment and M2-like polarization. Our results revealed the regulation of TAMs by cancer cells that underwent EMT.

Recently, studies have shown that miRNAs influenced the EMT process mainly by regulating the expression of EMT-related transcription factors [44]. Additionally, miRNAs have been extensively reported to be regulated by lncRNAs through various mechanisms. The lncRNA MPRL is known to enhance the drug resistance of tongue squamous cell carcinoma by disrupting the processing of mature miR-483-5p [30]. The lncRNA ATB has been reported to increase the expression of ZEB1 and ZEB2 by acting as ceRNA for the miR-200 family, inducing EMT in hepatocellular carcinoma cells [45]. The lncRNA Uc.283 + A promotes cancer progression by blocking the binding of DGCR8 and pri-miR-195 [46]. In our study, we demonstrated that *LINC00543* blocked the maturation of miR-506-3p by inhibiting the transport function of XPO5, which regulated FOXQ1-mediated EMT.

Our study provides solid results to support the idea that *LINC00543* remodels the TME in CRC and regulates EMT in CRC by modulating the pre-miR-506-3p/FOXQ1 axis. This study extends our understanding of the molecular mechanism of colorectal cancer metastasis to help develop novel therapeutic strategies in the future.

## Supplementary Information

**Additional file 1: Table S1.** Relationship between LINC00543 and clinicopathological features of CRC patients(n=40). **Table S2.** The sequences of the primers for quantitative RT-qPCR. **Table S3.** The Sequences of siRNAs, Plasmid. **Table S4.** The Sequences of LINC00543 segmental antisense nucleotide fragment biotin probes. **Fig. S1.** LINC00543 is highly expressed in CRC tissues and is associated with poor prognosis of CRC patients. a. ISH analysis of the expression of LINC00543 in CRC tissues. LINC00543 is demonstrate as a long non-coding RNA without encoding proteins ability on chromosome 13 through online websites b. pubmed (https://pubmed.ncbi.nlm.nih.gov/), c. LnCAR (https://lncar.renlab.org/), and d. CPC2 (http://cpc2.gao-lab.org/). **Fig. S2.** LINC00543 promotes the invasion and metastasis of CRC cells. The construction of LINC00543 knockdown and overexpression stably transfected cell lines. a. The knockdown efficiencies of Si-LINC00543A, Si-LINC00543B and Si-LINC00543C in SW620 cells. b. The knockdown efficiencies of SH-LINC00543A and SH-LINC00543B in cells. c. The transfection efficiency of LINC00543 in SW480 cells. d. The Ki67 score of NC and SH cells. **Fig. S3.** LINC00543 regulates the EMT of CRC cells. The expression levels of E-cadherin, N-cadherin, and Vimentin in NC group and SH group nude mice. **Fig. S4.** LINC00543 regulates EMT by upregulating FOXQ1 in CRC cells. The expression levels of EMT-related transcription factors in SH and LINC00543 cells. **Fig. S5.** The Polysome analysis of SH cells. **Fig. S6.** LINC00543 indirectly regulates FOXQ1 by downregulating miR-506-3p. The interaction of LINC00543 with miR-506-3p and Pre-miR-506-3p were predicted through online prediction website. a. The interaction of LINC00543 with miR-506-3p was predicted through online prediction website STARBASE (https://starbase.sysu.edu.cn/). b. The interaction of LINC00543 with miR-506-3p was predicted through online prediction website LncBASE (https://diana.e-ce.uth.gr/lncbasev3/interactions). c. The LINC00543 and Pre-miR-506-3p interaction were predicted through bioinformatics tool miRanda. **Fig. S7.** LINC00543 inhibits the transportation of pre-miR-506-3p. a. The relative expression levels of miR-506-3p, Pre-miR-506-3p, and Pri-miR-506-3p were detected in LINC00543 cells. b. RAP were applied to analyze the interaction between LINC00543 and Pre-miR-506-3p. c. The expression of Pre-miR-506-3p in the nucleus and cytosol of LINC00543 cells. d. RIP analysis of XPO5 interaction with Pre-miR-506-3p in LINC00543 cells. e. RIP analysis of XPO5 interaction with LINC00543 in LINC00543 cells. f. Relative expression of XPO5 in SH and LINC00543 cells. g. Agarose gel electrophoresis analysis of regions of LINC00543 combination with Pre-miR-506-3p. h. RAP analysis of the interaction of four LINC00543 segments with Pre-miR-506-3p. i. RIP analysis of the interaction of Pre-miR-506-3p and LINC00543 segments in incubated of XPO5 cells. j and k. Analysis of the interactions of LINC00543, XPO5 and Pre-miR-506-3p in cells incubated with antisense nucleotide biotin probes of the 3 binding regions. **Fig. S8.** LINC00543 promotes macrophage recruitment and M2 polarization through regulating CCL2 expression. Transwell assays were used to verify the relationship between the expression of LINC00543 and macrophage recruitment. **Fig. S9.** LINC00543 promotes the tumorigenesis of CRC and the M2 polarization of macrophages in vivo. a. The expression of Ki67 in THP1+NC group and THP1+SH group. b. The expression of CD163 and foxq1 in THP1+NC group and THP1+SH group.

## Data Availability

All the data and material are available at the Journal of translational medicine’s website.
