# Supplementary material for: LINC00543 promotes colorectal cancer metastasis by driving EMT and inducing the M2 polarization of tumor associated macrophages
Source: J Transl Med. 2023 Feb 25;21:153. doi: 10.1186/s12967-023-04009-6 (PMC9960415; doi:10.1186/s12967-023-04009-6)
Supplement: Supplementary file 1 — Additional file 1: Table S1. Relationship between LINC00543 and clinicopathological features of CRC patients(n=40). Table S2. The sequences of the primers for quantitative RT-qPCR. Table S3. The Sequences of siRNAs, Plasmid. Table S4. The Sequences of LINC00543 segmental antisense nucleotide fragment biotin probes. Fig. S1. LINC00543 is highly expressed in CRC tissues and is associated with poor prognosis of CRC patients. a. ISH analysis of the expression of LINC00543 in CRC tissues. LINC00543 is demonstrate as a long non-coding RNA without encoding proteins ability on chromosome 13 through online websites b. pubmed (https://pubmed.ncbi.nlm.nih.gov/), c. LnCAR (https://lncar.renlab.org/), and d. CPC2 (http://cpc2.gao-lab.org/). Fig. S2. LINC00543 promotes the invasion and metastasis of CRC cells. The construction of LINC00543 knockdown and overexpression stably transfected cell lines. a. The knockdown efficiencies of Si-LINC00543A, Si-LINC00543B and Si-LINC00543C in SW620 cells. b. The knockdown efficiencies of SH-LINC00543A and SH-LINC00543B in cells. c. The transfection efficiency of LINC00543 in SW480 cells. d. The Ki67 score of NC and SH cells. Fig. S3. LINC00543 regulates the EMT of CRC cells. The expression levels of E-cadherin, N-cadherin, and Vimentin in NC group and SH group nude mice. Fig. S4. LINC00543 regulates EMT by upregulating FOXQ1 in CRC cells. The expression levels of EMT-related transcription factors in SH and LINC00543 cells. Fig. S5. The Polysome analysis of SH cells. Fig. S6. LINC00543 indirectly regulates FOXQ1 by downregulating miR-506-3p. The interaction of LINC00543 with miR-506-3p and Pre-miR-506-3p were predicted through online prediction website. a. The interaction of LINC00543 with miR-506-3p was predicted through online prediction website STARBASE (https://starbase.sysu.edu.cn/). b. The interaction of LINC00543 with miR-506-3p was predicted through online prediction website LncBASE (https://diana.e-ce.uth.gr/lncbasev3/interactions). c [file 12967_2023_4009_MOESM1_ESM.pdf]

LINC00543 promotes colorectal cancer metastasis by driving EMT and inducing the M2 polarization of tumor associated macrophages

Jinsen Zheng[1,2,3,4]+, Rongzhang Dou[1,2,3,4]+, Xinyao Zhang[1,2,3,4]+, Bo Zhong[1,5], Chenggang Fang[5,] Qian Xu[1,2,3,4], Ziyang Di[1,2,3,4], Sihao Huang[1,2,3,4], Zaihuan Lin[1,2,3,4], Jialin Song[1,2,3,4], Shuyi Wang[1,2,3,4] and Bin Xiong[1,2,3,4]

1. Department of Gastrointestinal Surgery, Zhongnan Hospital of Wuhan University, Wuhan, Hubei, 430071, China.

2. Department of Gastric and Colorectal Surgical Oncology, Zhongnan Hospital of Wuhan University, Wuhan, Hubei, 430071, China.

3. Hubei Key Laboratory of Tumor Biological Behaviors, Wuhan, Hubei, 430071, China.

4. Hubei Cancer Clinical Study Center, Wuhan, Hubei, 430071, China.

5. Medical Research Institute of Wuhan University, Wuhan University, Wuhan, China

+ Jinsen Zheng, Rongzhang Dou, and Xinyao Zhang contributed equally to this work.

Supplemental Methods

Cell culture and Reagents

Five human CRC cell lines (HCT116, SW620, SW480, HT-29, and DLD1) and the human normal colon epithelial cell line NCM460 were purchased from the Chinese Academy of Sciences in Shanghai. The cells were cultured in RPMI 1640 (Gibco, USA) containing 10% fetal bovine serum (Gibco, USA) at 37 °C in a humidified atmosphere with 5% CO2. THP-1 cells (1×106) were incubated with 100 ng/mL PMA (Sigma-Aldrich, USA) for 24 h. Macrophages were co-cultivated with CRC cell lines using the non-contact co-culture transwell system (Corning, USA) for 48 h. Then macrophages or CRC cells were harvested for further analyses.

RNA extraction and quantitative reverse transcription PCR (RT-qPCR)

RNA isolation and quantitative real-time PCR (RT-qPCR). According to the manufacturer's instructions, the Trizol Reagent was used to isolate the total RNA from gastric cancer cells, adipocytes or tissues. Measure RNA concentration and quantify 1ug RNA for reverse transcription using the PrimeScript™ RT reagent kit (Toyobo, Osaka), then RT-qPCR was run on the Bio-Rad IQ5 Real PCR machine (Bio-Rad, USA) in a 96-well plate containing reaction mixture of pre-primer, post-primer, cDNA, SYBR-Green PCR Master Mix (Takara, Osaka). We used the $2^{\Delta\Delta Ct}$ method to calculate the relative expression. The primer sequences used in the study are presented in Table S2.

Western blot analysis

Cells or tissues were lysed in RIPA buffer and incubated with a protease inhibitor cocktail (Thermo Scientific, USA) at 4°C. The total proteins were separated by SDS-PAGE gels and

transferred to PVDF membranes (Millipore, USA). After blocking with 5% non-fat milk, the membranes were incubated with primary antibodies at 4°C overnight. The membranes were washed and incubated for 1h at room temperature with HRP-conjugated secondary antibodies. Proteins were detected using a Bio-Rad ChemiDoc XRS+System. Bio-Rad Image Lab software was used for densitometric analysis. The following primary antibodies were used: anti-ZEB1, anti-ZEB2, anti-SNAIL1, anti-SNAIL2 and anti-TWIST (1:1000; CST, USA); anti-FOXQ1 (1:1000; Proteintech, USA); anti-E-cadherin, anti-N-cadherin and anti-Vimentin (1:1000; CST, USA); anti-CD163, anti-Ki67 (1:1000; CST, USA); anti-GAPDH and anti-β-actin (1:5000; CST, USA). The images were analyzed by ImageJ software (NIH, USA).

Plasmid constructs, siRNAs, miRNAs, and transfections

The sequences of SiRNA targeting *LINC00543*, FOXQ1, and negative control (NC) were designed in GenePharma. miR-506-3p mimics and inhibitor were obtained from RiboBio Co. Ltd., China. According to the manufacturer's protocol, CRC cells were transfected using Lipofectamine 2000 (Invitrogen, USA). When cells were transfected after 48 to 72 hours, the transfection efficiency was determined through quantitative reverse transcription PCR (RT-qPCR). The sequences of SiRNAs were listed in Table S3. *LINC00543* plasmid vector and negative control were chemically synthesized in Genecopia (MD, USA). Stably transfected cells were derived from the parental cells by puromycin (Sigma-Aldrich, USA) selection. Cells treated above were plated for a functional assay or harvested for RNA and protein analyses.

Animal assays

All animal experiments were performed according to our institutions' guidelines for the use of laboratory animals and were approved by the Institutional Animal Care and ethical

committee of Zhongnan hospital of Wuhan University. For the subcutaneous models, the nude mice were divided into 5 randomized groups (n=6 per group). SW620-*LINC00543*-NC cells alone (1×10^7), SW620-*LINC00543*-SH cells alone (1×10^7), TAMs, *LINC00543*-NC and TAMs cells *LINC00543*-SH were subcutaneously injected into nude mice. For the spleen-injected liver metastasis models, the nude mice were divided into 2 groups (n=5 per group). in 200ul were subcutaneously injected into spleen of nude mice. After 30 days, the mice were euthanized and were necropsied to assess metastatic burden.

Transwell migration, invasion assay, and wound healing assay

Transwell chambers (8 μm pore size; Corning, USA) with or without Matrigel (Corning, USA) were used to determine the migration and invasion ability of CRC cells. The detailed steps of Transwell migration and invasion assay have changed compared with the methods previously described[1]. In total, 5×10^4 THP1 cells were suspended in 500 μl RPMI 1640 containing 5μg DIL and added to the upper chamber. Contents in the lower chamber were divided into eight groups (blank, NC, SH, SH+Anti-CCL2 antibody, blank, Vector, *LINC00543*, and LINC00543+Anti-CCL2ab) and were suspended in 750 μl RPMI 1640 containing 10% FBS. After 48 h of incubation, Matrigel and the cells remaining in the upper chamber were removed using cotton swabs. Cells on the lower surface of the membrane were fixed in 4% paraformaldehyde and stained with DAPI. Cells in 5 microscopic fields were counted and photographed.

A wound-healing assay was used to evaluate the ability of CRC cells. Cells were grown to 80–90% confluence in 24-well plates, and a wound was made by a plastic pipette tip across the cell surface. The remaining cells were washed three times in PBS to remove cellular debris and

incubated at 37 °C with serum-free medium. Migrating cells at the wound front were photographed after 24 h. All experiments were performed in triplicate. The area of the wound was measured with Image J software (NIH, USA).

HE staining, immunohistochemistry

Paraffin-Embedded Samples were taken for HE staining and immunohistochemistry. The antibodies used in immunohistochemistry as follows: anti-CD163, anti-Ki67 (1:1000; CST, USA), and anti-FOXQ1 (1:1000; Proteintech, USA). HE staining and immunohistochemistry were performed as our previously reported[1]. The expression levels were scored semiquantitatively based on staining intensity and distribution using the immunoreactive score (IRS) as described elsewhere. Briefly, Immunoreactive score (IRS) = SI (staining intensity) × PP (percentage of positive cells). SI was assigned as: 0 = negative; 1 = weak; 2 = moderate; 3 = strong. PP is defined as 0 = 0%; 1 = 0–25%; 2 = 25–50%; 3 = 50–75%; 4 = 75–100%.

Immunofluorescence (IF) Staining

E-cadherin, N-cadherin, and Vimentin in SH and *LINC00543* cells were determined by Immunofluorescence staining. Stably transfected cells were cultivated on glass coverslips in 24-well plates for 48h, and then the samples were fixed in 4% paraformaldehyde (PFA) for 40 min. After fixed, the cells were permeabilized with 0.25% Triton X-100 for 10 min at room temperature and blocked with 4% bovine serum albumin. The coverslips were incubated with primary antibodies anti-E-cadherin, anti-N-cadherin and anti-Vimentin (1:200 dilution, Cell Signaling Technology) overnight at 4 °C. After three times washings with phosphate-buffered saline (PBS), the coverslips were incubated in the fluorescent secondary antibody for 50min. Cellular nuclei were stained for 10 min with 4′,6-diamidino-2-phenylindole dihydrochloride

(DAPI; Life Technologies, Carlsbad, CA, USA). Cells were observed under an inverted microscope (Olympus BX53).

Fluorescence in situ Hybridization (FISH) and in situ hybridization Assay

The FISH assay was conducted to determine the subcellular localization of *LINC00543* in GC cells. On the basis of the instructions of LncRNA FISH Probe Mix (Boster Biological Technology Co, Ltd, China), cells were fixed in 4% PFA for 30 min at room temperature and then permeabilized with 0.5% Triton X-100 for 20 min at 4 °C. After the processes above, the cells were incubated with digoxigenin (DIG)-labeled *LINC00543* probe at 42°C for 2h. Subsequently, the sample was incubated with a hybridization solution at 42°C overnight. After three times washings with PBS, the nuclei were stained with DAPI. Finally, images were obtained using a fluorescence microscope (Olympus). The *LINC00543* expression in paraffin-embedded samples and xenograft tumor specimens of nude mice were examined through ISH. The expression levels of *LINC00543* were scored semiquantitatively based on staining intensity and distribution using the immunoreactive score (IRS) as described elsewhere. Briefly, Immunoreactive score (IRS) = SI (staining intensity) × PP (percentage of positive cells). SI was assigned as: 0 = negative; 1 = weak; 2 = moderate; 3 = strong. PP is defined as 0 = 0%; 1 = 0–25%; 2 = 25–50%; 3 = 50–75%; 4 = 75–100%.

Cytoplasmic and Nuclear Fraction

CRC cells were collected by centrifugation and resuspended in 800μL hypotonic buffer (10mM Heps, pH7.9, 1.5mM MgCl2, 10 mM KCl). Then Nonidet P-40 (3.5μL) was added to the sample. Samples were inverted a few times and spun at 3,000 rpm for 7 min. Supernatants (450μL; cytoplasmic fraction) were collected for processing. The precipitate (nuclear fraction)

was resuspended in 500μL hypotonic buffer and spun at 3,000 rpm for 2 min. This washing step was repeated three times. After removing the buffer following the last spin, the nuclear fraction was obtained.

Polysome Fractionation assay

The detailed steps of Polysome Fractionation assay were performed according to the methods previously described [2]. In brief, the stably transfected cells were incubated with cycloheximide (Calbiochem; 100 μg/ml, 15 min) followed by preparation of cytoplasmic lysates using PEB. Lysates were size-fractionated by centrifugation through 10–50% linear sucrose gradients and 12 fractions were collected for further analysis. RNA in each fraction was monitored by measuring absorbance at 254 nm using a spectrophotometer was analyzed by RT-qPCR analysis and plotted as a percentage of the specific mRNA in each fraction relative to the total amount of that mRNA in the gradient.

Luciferase reporter assay

For Luciferase reporter assay, a 2000-bp DNA fragment upstream from the FOXQ1 promoter was cloned into GPL3-Basic plasmid (GenePharma, China). Lipofectamine 2000 was used to transfected GPL3-Basic luciferase reporters, negative control and renilla luciferase reporter vector pRL-SV40 (Promega, USA) into SW620-NC, SW620-SH, SW480-vector and SW480-OE cells, respectively. The total cell lysates were harvested 48 h after transfection, and luciferase activities were determined using Dual-Luciferase reporter system (Promega, USA) according to the manufacturer's instructions.

Enzyme-linked immunosorbent assay (ELISA) and Agarose gel electrophoresis

The CCL2 content in the supernatant of cells culture fluids was measured by ELISA kit

(Bioswamp, China) according to manufacturer's protocol. DNA samples were separated with 0.5% agarose gel. Bio-Rad ChemiDoc XRS+System was used to observe the gel and photograph.

1. Wei C, Yang C, Wang S, Shi D, Zhang C, Lin X, et al. Crosstalk between cancer cells and tumor associated macrophages is required for mesenchymal circulating tumor cell-mediated colorectal cancer metastasis. *Molecular cancer* **18**,64 (2019).

2. Panda AC, Martindale JL, Gorospe M. Polysome Fractionation to Analyze mRNA Distribution Profiles. *Bio Protoc* **7**2017).

Supplemental Tables

Table S1. Relationship between *LINC00543* and clinicopathological features of CRC patients(n=40).

		***LINC00543* expression**			
**Parameters**	**n（%）**	**< median**	**≥ median**	**value**	**P value**
**Gender**					
Male	23（57.5）	11	12	0.1023	0.74908

Female	17 (42.5)	9	8		
**Age，years**					
< 60	17 (42.5)	8	9	0.1023	0.74908
≥60	23 (57.5)	12	11		
**Tumor size**					
< 5	23 (57.5)	10	13	0.92072	0.33729
≥5	17 (42.5)	10	7		
**Tumor grade**					
Poor	6 (15.0)	1	5	1.76471	0.18404
Moderate/well	34 (85.0)	19	15		
**Lymphovascular invasion**					
Absence	22 (55.0)	15	7	6.46465	0.011*
Presence	18 (45.0)	5	13		

**Perineural invasion**					
Absence	10 (25.0)	10	0	13.3333	0.00026***
Presence	30 (75.0)	10	20		
**Tumor invasion**					
T1-2	8 (20.0)	7	1	3.91	0.04811*
T3-4	32 (80.0)	13	19		
**Lymph node metastasis**					
N0-1	31 (77.5)	20	11	9.17563	0.00245**
N2-3	9 (22.5)	0	9		
**TNM stage[a]**					
I/II	22 (55.0)	18	4	20.14	0.00004***
III	13 (32.5)	2	11		

IV	5 (12.5)	0	5	

Notes: [a]The 8th edition of the AJCC Cancer Staging Manual. Two-way ANOVA analysis; * $p < 0.05$, ** $p < 0.01$, *** $p < 0.001$.

Table S2. The sequences of the primers for quantitative RT-qPCR.

Gene name	Primer	Sequences (5′ to 3′)
LINC00543	FORWARD	ACCAGGCCCTCCTAGAAACT
	REVERSE	TTCCTGGGCGTGATCACAAG
E-cadherin	FORWARD	ATTTTTCCCTCGACACCCGAT
	REVERSE	TCCCAGGCGTAGACCAAGA
N-cadherin	FORWARD	TGCGGTACAGTGTAACTGGG
	REVERSE	GAAACCGGGCTATCTGCTCG
Vimentin	FORWARD	AGTCCACTGAGTACCGGAGAC
	REVERSE	CATTTCACGCATCTGGCGTTC
GADPH	FORWARD	CTGTTCGACAGTCAGCCGCATC
	REVERSE	GCGCCCAATACGACCAAATCCG
ZEB1	FORWARD	CAGCTTGATACCTGTGAATGGG
	REVERSE	TATCTGTGGTCGTGTGGGACT
ZEB2	FORWARD	AGGAGCAGGTAATCG
	REVERSE	TGGGCACTCGTAAGG
SNAIL1	FORWARD	TCGGAAGCCTAACTACAGCGA

	REVERSE	AGATGAGCATTGGCAGCGAG
SNAIL2	FORWARD	CGAACTGGACACACATACAGTG
	REVERSE	CTGAGGATCTCTGGTTGTGGT
TWIST	FORWARD	AATTCAAAGAAACAGGGCGTG
	REVERSE	GCACGACCTCTTGAGAATGC
FOXQ1	FORWARD	TAAAGACTCAGCCAGTGGGC
	REVERSE	CGTCTGCGAAGGTGTACTCA
Pri-miR-506-3p	FORWARD	GCCACCACCATCAGCCATACTATG
	REVERSE	ACTCGTCATTACCAATGCCACCAC
Pre-miR-506-3p	FORWARD	GCCACCACCATCAGCCATAC
	REVERSE	GCCACCACAAATGTTGTCCATG
miR-506-3p	FORWARD	TCTGAGTAGAGTCGTATCCAGT
	REVERSE	AATTGCACTGGATACGACTCTACTCA
	RT Primer	GTCGTATCCAGTGCGTGTCGTGGAGTCGGC
XPO5	FORWARD	GAGGCTCTGTCAGGTGTTGT
	REVERSE	ATTTCATGCCTGAAGAGGGCT
CCL2	FORWARD	GCCACCACCATCAGCCATAC
	REVERSE	GGTGGAGCCATCCTGACTTC
18S	FORWARD	CGGACAGGATTGACAGATTGATAGC
	REVERSE	TGCCAGAGTCTCGTTCGTTATCG
CD206	FORWARD	GGGTTGCTATCACTCTCTATGC
	REVERSE	TTTCTTGTCTGTTGCCGTAGTT

CD163	FORWARD	TTTGTCAACTTGAGTCCCTTCAC
	REVERSE	TCCCGCTACACTTGTTTTCAC
IL-10	FORWARD	TTAAGGGTTACCTGGGTTGC
	REVERSE	CTGGGTCTTGGTTCTCAGCTT
Arg-1	FORWARD	TGGACAGACTAGGAATTGGCA
	REVERSE	CCAGTCCGTCAACATCAAAACT
IL-12	FORWARD	CCTTGCACTTCTGAAGAGATTGA
	REVERSE	ACAGGGCCATCATAAAAGAGGT
IL-1β	FORWARD	TGAACTGAAAGCTCTCCACC
	REVERSE	CTGATGTACCAGTTGGGGAA
HLA-DR	FORWARD	TCTGGCGGCTTGAAGAATTTG
	REVERSE	GGTGATCGGAGTATAGTTGGAGC
CD86	FORWARD	TGTAACAGGGACTAGCACAGACA
	REVERSE	CAGCACCACTGGGGATCCAT
U6	FORWARD	CTCGCTTCGGCAGCACA
	REVERSE	AACGCTTCACGAATTTGCGT
	RT Primer	AACGCTTCACGAATTTGCGT
β-Actin	FORWARD	ACAGAGCGTGGCTACACATT
	REVERSE	TCCAGGGCAACATAGCACAG

Table S3. The Sequences of siRNAs, Plasmid.

Gene name	Sequences (5′ to 3′)

	Si#NC：UUCUCCGAAGGUGUCACGUTT
	Si#A：GCAAAUCAGGAAGUGUCUUTT
	Si#B：GCUUGGCGUCUUCAGGUAUTT
LINC00543	Si#C：GCGAAUCUCUCCACGGAAUTT
	SH#NC：TTCTCCGAACGTGTCACGT
	SH#A：GGACATCACACCATTCCAAAG
	SH#B：GCGAATCTCTCCAGGGAATCC
FOXQ1	Si#NC：UUCUCCGAAGGUGUCACGUTT<br>Si：GCCCAGGCUUCGUCUUAUUTT

Table S4. The Sequences of *LINC00543* segmental antisense nucleotide fragment biotin probes.

Probe	Target Sequences (5′ to 3′)
Control	CAAACGGCGGATTGACCGTAATGGGATAGGTCACGTTGGTGTAGATGGGCGCATCGTAAC

LINC00543A	TAATCTACTCCTTTCCACTCTCTCGGTGGCGCTGTGGCTCTTAAA
LINC00543B	AAGGGATTTGATTTTCAAAAGGCGGAACGGGAGCG
LINC00543C	TGTCCTTATACCTGAAGACGCCAAGCTTTTCAAAAGGTGC
LINC00543D	GCGGATCTACCCCAGCCATTAGAGCAGATAGAGCT
LINC00543E	CAAATGATCATGCCATGTGAAGCTGGTGGGTAAGTTTCTAGGAGG

Supplemental Figures and Legends

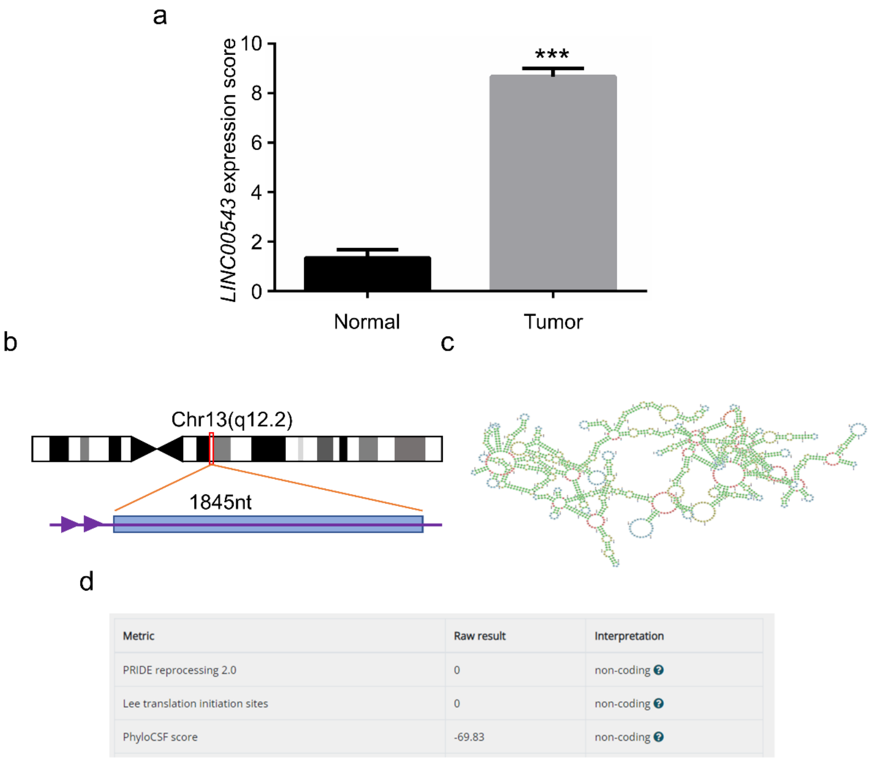

Fig.S1 ***LINC00543*** **is highly expressed in CRC tissues and is associated with poor prognosis of CRC patients.**

a. ISH analysis of the expression of *LINC00543* in CRC tissues. *LINC00543* is demonstrate as a long non-coding RNA without encoding proteins ability on chromosome 13 through online websites **b.** pubmed (https://pubmed.ncbi.nlm.nih.gov/), **c.** LnCAR (https://lncar.renlab.org/), and **d.** CPC2 (http://cpc2.gao-lab.org/).

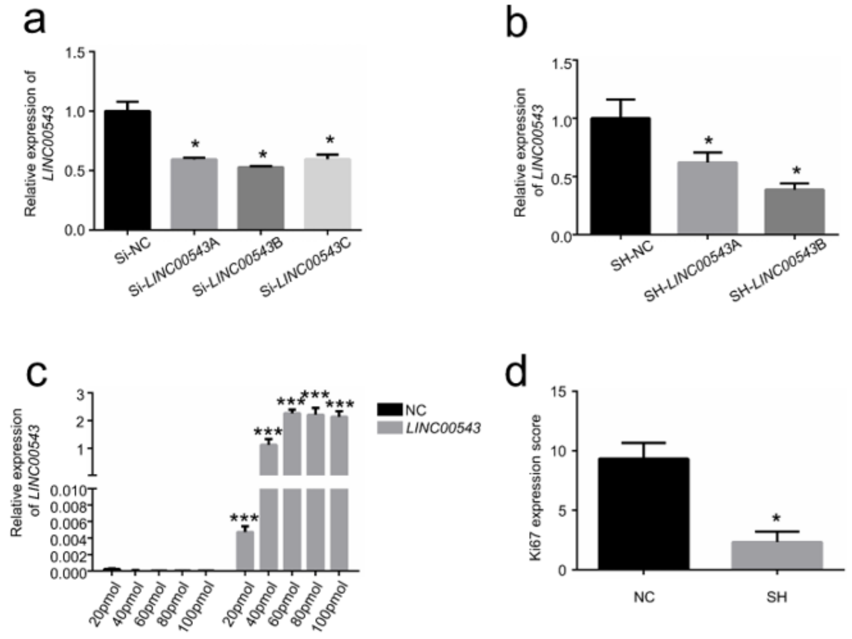

Fig.S2 ***LINC00543*** **promotes the invasion and metastasis of CRC cells.**

The construction of *LINC00543* knockdown and overexpression stably transfected cell lines. **a.** The knockdown efficiencies of Si-*LINC00543*A, Si-*LINC00543*B and Si-*LINC00543*C in SW620 cells. **b.** The knockdown efficiencies of SH-*LINC00543*A and SH-*LINC00543*B in cells. **c.** The transfection efficiency of *LINC00543* in SW480 cells. **d.** The Ki67 score of NC and SH cells.

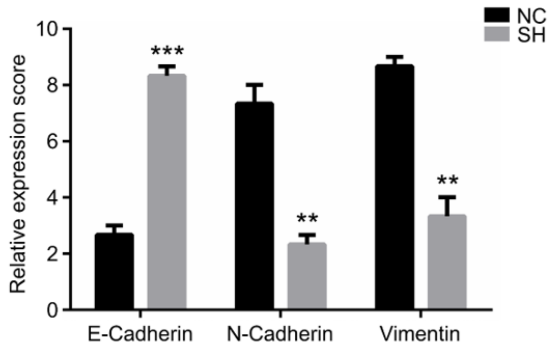

Fig.S3 ***LINC00543*** **regulates the EMT of CRC cells**.

The expression levels of E-cadherin, N-cadherin, and Vimentin in NC group and SH group nude mice.

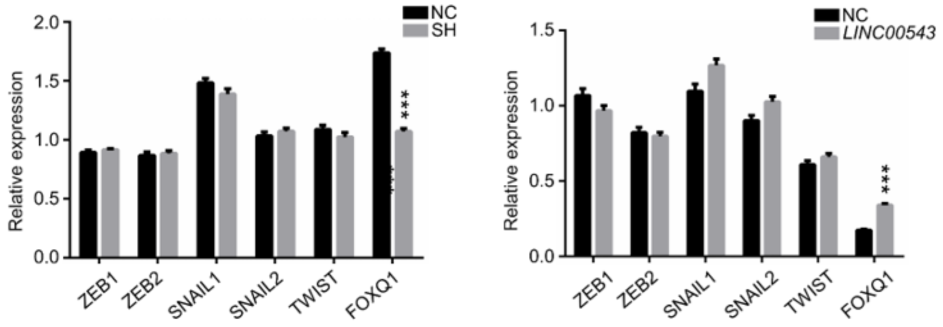

Fig.S4 ***LINC00543*** **regulates EMT by upregulating FOXQ1 in CRC cells**.

The expression levels of EMT-related transcription factors in SH and *LINC00543* cells.

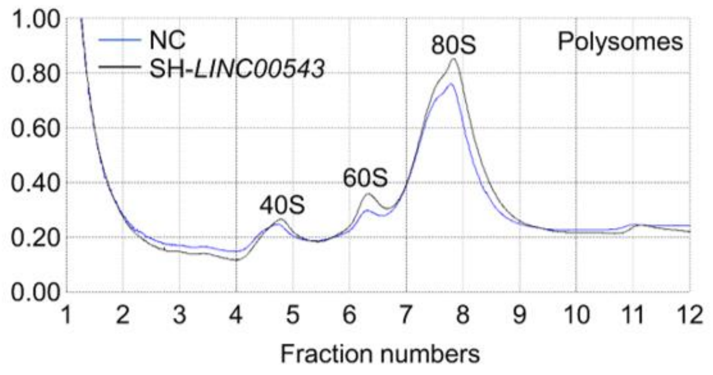

Fig.S5 The Polysome analysis of SH cells.

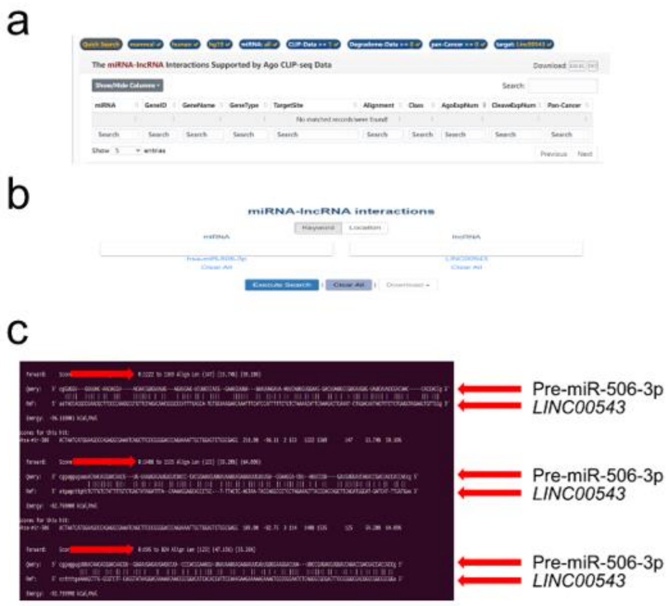

Fig.S6 *LINC00543* indirectly regulates FOXQ1 by downregulating miR-506-3p.

The interaction of *LINC00543* with miR-506-3p and Pre-miR-506-3p were predicted through online prediction website. **a.** The interaction of *LINC00543* with miR-506-3p was predicted through online prediction website STARBASE (https://starbase.sysu.edu.cn/). **b.** The interaction of *LINC00543* with miR-506-3p was predicted through online prediction website LncBASE (https://diana.e-ce.uth.gr/lncbasev3/interactions). **c.** The *LINC00543* and Pre-miR-506-3p interaction were predicted through bioinformatics tool miRanda.

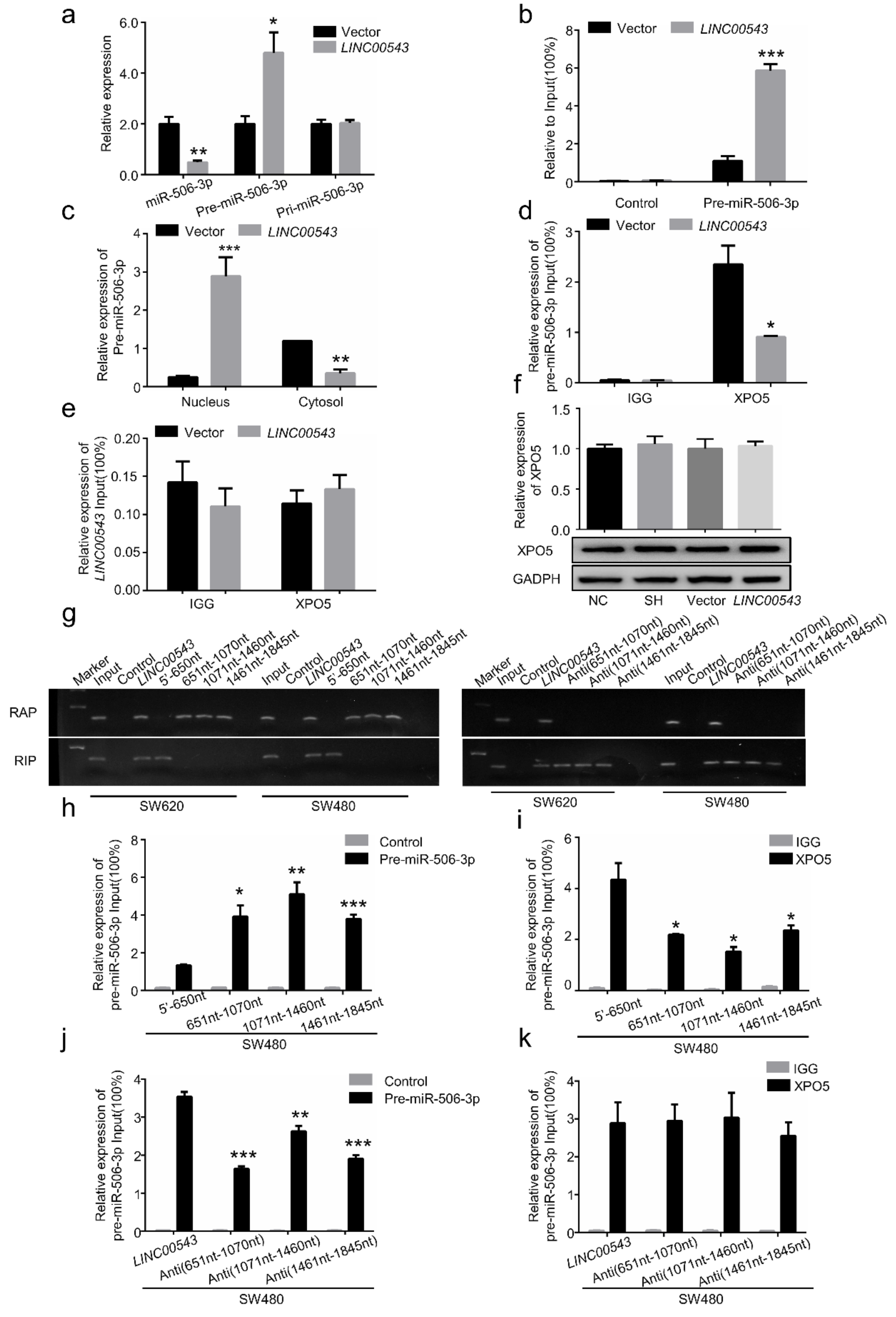

Fig.S7 *LINC00543* inhibits the transportation of pre-miR-506-3p.

a. The relative expression levels of miR-506-3p, Pre-miR-506-3p, and Pri-miR-506-3p were detected in *LINC00543* cells. **b.** RAP were applied to analyze the interaction between

LINC00543 and Pre-miR-506-3p. **c.** The expression of Pre-miR-506-3p in the nucleus and cytosol of *LINC00543* cells. **d.** RIP analysis of XPO5 interaction with Pre-miR-506-3p in LINC00543 cells. **e.** RIP analysis of XPO5 interaction with *LINC00543* in *LINC00543* cells. **f.** Relative expression of XPO5 in SH and *LINC00543* cells. **g.** Agarose gel electrophoresis analysis of regions of *LINC00543* combination with Pre-miR-506-3p. **h.** RAP analysis of the interaction of four *LINC00543* segments with Pre-miR-506-3p. **i.** RIP analysis of the interaction of Pre-miR-506-3p and *LINC00543* segments in incubated of XPO5 cells. **j** and **k.** Analysis of the interactions of *LINC00543*, XPO5 and Pre-miR-506-3p in cells incubated with antisense nucleotide biotin probes of the 3 binding regions.

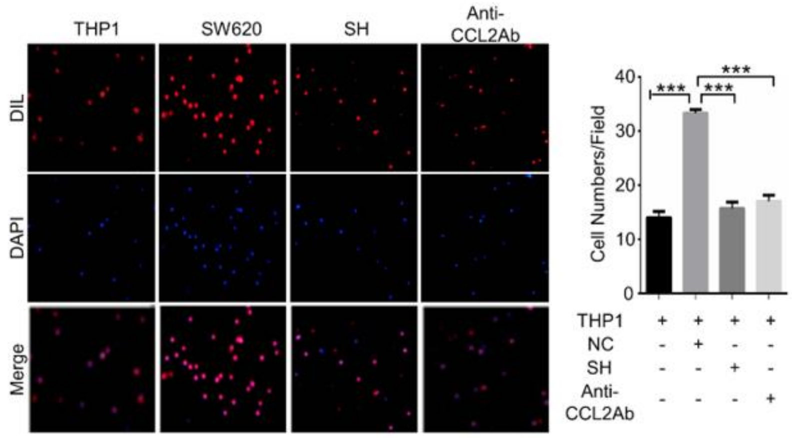

Fig.S8 *LINC00543* promotes macrophage recruitment and M2 polarization through regulating CCL2 expression.

Transwell assays were used to verify the relationship between the expression of *LINC00543* and macrophage recruitment.

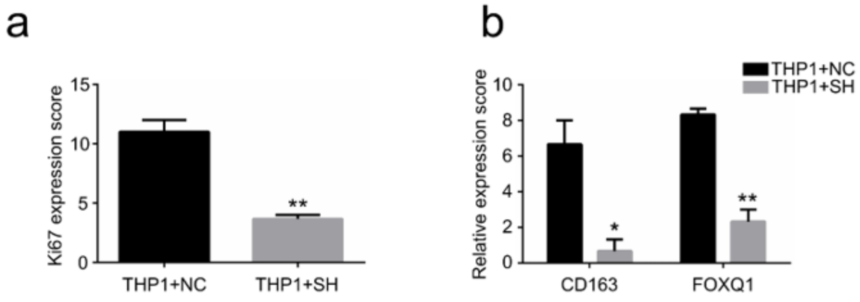

Fig.S9 *LINC00543* promotes the tumorigenesis of CRC and the M2 polarization of macrophages *in vivo*.

a. The expression of Ki67 in THP1+NC group and THP1+SH group. **b.** The expression of CD163 and foxq1 in THP1+NC group and THP1+SH group.
